# A Targeted Approach by High Resolution Mass Spectrometry to Reveal New Compounds in Raisins

**DOI:** 10.3390/molecules25061281

**Published:** 2020-03-12

**Authors:** Danilo Escobar-Avello, Alexandra Olmo-Cunillera, Julián Lozano-Castellón, María Marhuenda-Muñoz, Anna Vallverdú-Queralt

**Affiliations:** 1 Department of Nutrition, Food Science and Gastronomy, School of Pharmacy and Food Sciences and XaRTA, Institute of Nutrition and Food Safety (INSA-UB), University of Barcelona, 08921 Santa Coloma de Gramenet, Spain; daniescobar01@gmail.com (D.E.-A.);; 2 Consorcio CIBER, M.P. Fisiopatología de la Obesidad y la Nutrición (CIBERObn), Instituto de Salud Carlos III (ISCIII), 28029 Madrid, Spain

**Keywords:** polyphenols, dried fruit, *Vitis vinifera*, LC-LTQ-Orbitrap, condensed tannins

## Abstract

Raisins are dried grapes mostly obtained from cultivars of *Vitis vinifera* L. and are extensively consumed worldwide. They are rich in bioactive compounds such as polyphenols, which are associated with a broad range of health benefits. The aim of the present study was to compare the phenolic profiles of three different raisin varieties (Thompson seedless, Muscat, and sultanas). Total polyphenols (TPs) were evaluated by the Folin–Ciocalteu (F–C) assay and significant differences were observed among all raisin varieties. Furthermore, liquid chromatography coupled with electrospray ionization hybrid linear ion trap quadrupole-Orbitrap-mass spectrometry (LC/ESI-LTQ-Orbitrap-MS) was employed for the comprehensive identification of phenolic constituents. A total of 45 compounds were identified, including hydroxybenzoic and hydroxycinnamic acids, flavanoids, flavonoids, flavonols, flavones, and stilbenoids. The three varieties of raisins showed a similar phenolic profile, although the highest number of phenolic compounds was identified in Muscat raisins owing to the proanthocyanidins extracted from their seeds, while stilbenoids were not detected in the Thompson variety.

## 1. Introduction

Raisins are dried grapes mostly obtained from cultivars of *V. vinifera* L. and are extensively consumed worldwide. Although sometimes perceived as unhealthy because of their high sugar content, raisins represent an important source of bioactive compounds, including fiber and polyphenols, which may benefit human health [1].

Interest in phenolic compounds is growing as scientific research brings further evidence of their beneficial effects. The major polyphenols found in raisins are phenolic acids (caftaric and coutaric acid) and flavonols (quercetin and kaempferol glycosides, and rutin) [2,3,4,5]. Anthocyanins have also been identified in dark raisins [6,7,8]. Flavan-3-ols and procyanidins have been detected in Turkish raisins [7].

Scientific literature suggests that a long-term ingestion of foods rich in polyphenols protects against certain health conditions, such as cardiovascular and neurodegenerative diseases, cancer, and diabetes [9,10]. The antioxidant, immunomodulatory, and scavenging properties of catechins have been associated with the protection of neurons and the inhibition of the neurotoxic effects of the beta-amyloid protein, thus helping to ward off Alzheimer’s disease and dementia. Flavanoids may decrease cancer risk by the neutralization of free radicals. The risk of cardiovascular disease is lowered by flavonoids, which prevent cholesterol oxidation, while anthocyanins are associated with the prevention and management of type 2 diabetes. Moreover, it is hypothesized that polyphenols promote the growth of beneficial gut microbiota [11].

The phenolic content of raisins is affected by multiple factors, including genetics (for instance, the presence or not of seeds and grape color) [12], environmental growing conditions, and the drying process (for instance, degree of temperature and UV-B radiation) [13,14,15]. Moreover, the type of analytical extraction procedure can also influence the phenolic profile detected [4,16].

High-performance liquid chromatography (HPLC) is one of the most commonly applied methods to identify and quantify polyphenols in food [17], and UV/vis and DAD detectors are more frequently used than mass spectrometry (MS) because they are less expensive, comparably convenient to operate, and suitable for routine analysis [18]. However, HPLC coupled with MS allows a more accurate identification [17] and increases the range of phenolic compounds detected in a sample [19].

Although the phenolic content of raisins has been widely studied, the available data could be updated using recently developed techniques offering more specific identification. Orbitrap technology stands out for its high mass accuracy and resolution, which provides more information about compounds present in a mixture [20]. This is particularly useful for genomics, proteomics, and metabolomics studies analyzing highly complex matrices. This approach has been successfully used to identify phenolic compounds in grape cane extracts [21], wine extracts [22,23], culinary herbs and spices [24], tomatoes [25], and cacao and chocolate [26]. Therefore, the aim of this study was to identify the polyphenols in three types of commonly consumed raisins (Thompson seedless, Muscat, and sultanas) by liquid chromatography hybrid linear ion trap quadrupole-Orbitrap-mass spectrometry (LC-LTQ-Orbitrap-MS) analysis.

## 2. Results and Discussion

### 2.1. Phenolic Profile of Commercial Raisins

Raisins have been reported as a good source of polyphenols with potential human health benefits and effects against different diseases [1].

The Folin–Ciocalteu (F–C) method gave results indicating a high polyphenol content in the three varieties of raisins analyzed. Overall, total polyphenol (TP) levels ranged from 327 to 664 mg GAE/100 g, with the highest amount in Muscat raisins (664 ± 15 mg GAE/100 g), followed by Thompson seedless (472 ± 7.7 mg GAE/100 g) and then sultanas (327 ± 3.9 mg GAE/100 g). Furthermore, significant differences (*p* < 0.05) in TP content were observed among all raisin varieties. These values are greater than those reported in raisins by Fabani et al. [5] (136–201 mg GAE/100 g) and Chiou et al. [27] (151–246 mg GAE/100 g), but comparable to other studies [12,16,28]. Differences in reported TP levels could be partially explained by different water content [29], environmental growing conditions [28], raisin variety [12], the drying process [14], and the choice of extraction solvents [16] or analytical methods [4].

A targeted analysis of phenolic compounds in raisins was performed using LC-LTQ-Orbitrap-MS. For the **45** compounds identified, accurate mass measurements of the [M − H]^−^ ion, molecular formula (MF), and MS^2^ fragment ions used for identification are listed in Table 1. The most represented classes of phenolic compounds in the extracts were phenolic acids (hydroxybenzoic and hydroxycinnamic acids) and flavonoids (flavanols, flavanones, and flavonols). Some stilbenes were also identified. Although many of these compounds have been previously detected in raisins [2,5,7,12,13,16,27,28], to the best of our knowledge, six have never been identified in these raisin varieties.

#### 2.1.1. Phenolic Acids and Derivatives

Phenolic acids are present in almost all plant-derived foods and are the main polyphenols in raisins. Their biological activities include effects against aging, diabetes, cardiovascular disease, and cancer [30].

Seventeen phenolic acids, hydroxycinnamic and hydroxybenzoic, were identified in the three raisin varieties (Table 1). The spectra generated by hydroxycinnamic and hydroxybenzoic acids showed the deprotonated molecule [M − H]^−^ and the characteristic loss of CO_2_ [M − H—44]^−^ [25,31], whereas those of phenolic acid-*O*-hexosides revealed the typical cleavage of the sugar moiety [M − H—162]^−^ [31].

Nine hydroxybenzoic acids—gallic acid (*m/z* 169.0135), protocatechuic acid-*O*-hexoside isomer 1 (*m/z* 315.0709), protocatechuic acid (*m/z* 153.0186), and 2-hydroxybenzoic acid (*m/z* 137.0238)—were detected in all raisin varieties. Furthermore, gallic acid was identified by comparison with its pure standard. On the other hand, two galloyl-hexoside (*m/z* 331.0657; *m/z* 331.0656) and hydroxybenzoic acid hexoside (*m/z* 299.0761), were only identified by MS^2^ in Muscat and sultana varieties, while 4-hydroxybenzoic acid (*m/z* 137.0238) was detected in Thompson seedless and sultanas varieties. Lastly, gallic acid ethyl ester (*m/z* 197.0446) was identified in Muscat raisins and confirmed by comparison with the available standard. Gallic, protocatechuic, and 2-hydroxybenzoic acid have been previously found in Thompson raisins [12]. Interestingly, in other studies, protocatechuic acid has been found in raisins and not in grapes [3,4]. The presence of protocatechuic acid in raisins could be attributed to the drying process. On the other hand, some hydroxybenzoic acids hexosides have been previously reported in red (Cardinal) and white (Sabel) dried grapes [32].

A total of eight hydroxycinnamic acids were identified in the three raisin varieties. Two dihydroxy cinnamic acid isomers 1 and 2 (*m/z* 179.0342; *m/z* 179.0343), caftaric acid isomer 2 (*m/z* 311.0395), coutaric acid (*m/z* 295.0447), and ferulic acid-*O*-hexoside (*m/z* 355.1020) were identified in all raisin varieties studied. Coutaric acid was tentatively identified based on the loss of the tartaric acid [M—H—132]^—^ and the presence of a fragment at *m/z* 163, attributed to a coumaric acid molecule [21,33,34]. Additionally, the dihydroxycinnamic acid isomer 2 was identified by comparison with its pure standard. On the other hand, caftaric acid isomer 1 (*m/z* 311.0394) and fertaric acid (*m/z* 325.0550) were only identified by MS^2^ in Muscat and sultana varieties. Fertaric acid showed product ions at *m/z* 193 and *m/z* 149, attributed to ferulic acid and tartaric acid, respectively [33]. Lastly, coumaric acid-*O*-hexoside (*m/z* 325.0914) was detected in Thompson and Muscat varieties.

Most of the detected phenolic acids have already been reported in raisins [3,5,12,16,27,32], but to the best of our knowledge, coumaric and ferulic acid hexoside derivatives have not been detected in the studied varieties.

#### 2.1.2. Flavanols (Proanthocyanidins)

Proanthocyanidins (PAs), also referred to as condensed tannins, are the most abundant plant-derived polyphenols. PAs exert several pharmacological effects, including antioxidant, antimicrobial, anti-cancer, and cardio-protective effects [35]. They are also useful for bio-based material design [36,37,38].

Fourteen flavanols were tentatively identified in the extract of Muscat raisins, probably because these contain seeds, which are known to have high levels of PAs [34]. The most common classes of PAs consist of subunits of catechin, epicatechin, and their gallic acid esters (mainly B-type oligomers). Flavan-3-ols catechin (*m/z* 289.0707) and epicatechin (*m/z* 289.0705) were also detected in Muscat and sultana varieties, and were confirmed after comparison with their standards.

**Figure 1:**
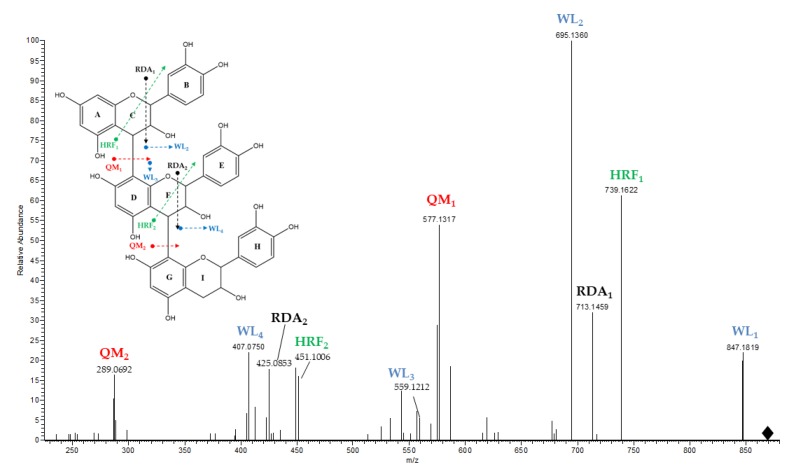
Fragmentation pathways of procyanidins (DP1–DP3) in negative mode electrospray ionization (ESI)-mass spectrometry (MS)^n^. Water loss (WL), Quinone methide fission (QM), heterolytic ring fission (HRF), and retro-Diels-Alder (RDA) mechanism.

Five B-type procyanidin dimers (*m/z* 577.1330; *m/z* 577.1329; *m/z* 577.1328; *m/z* 577.1326; *m/z* 577.1334) and two B-type procyanidin trimers (*m/z* 865.1942; *m/z* 865.1944) were detected in Muscat raisins. Procyanidins showed typical fragmentation pathways including quinone methide (QM) fission, heterolytic ring fission (HRF), and the retro-Diels-Alder (RDA) mechanism (Figure 1). The mass spectra of these compounds have been extensively described in previous studies [21,34].

Catechin, epicatechin, and procyanidin (B1 and B2) have been identified in Turkish raisins [7]. Nevertheless, Karadeniz et al. [2] reported that procyanidins and flavan-3-ols were degraded when grapes were transformed into raisins. Similarly, Fabani et al. [5] detected the procyanidin dimer only in fresh grape extracts and suggested it underwent degradation during the sun-drying process, probably owing to enzymatic oxidation. In our work, procyanidin dimers and trimers, previously reported in grape seeds [34], were identified exclusively in the Muscat raisins, the only variety studied bearing seeds. PAs from grape seeds have shown broad pharmacological and therapeutic health effects against cancer, obesity, diabetes mellitus, and cardiovascular disease, which are related to oxidative stress and inflammatory processes [39].

**Figure 2:**
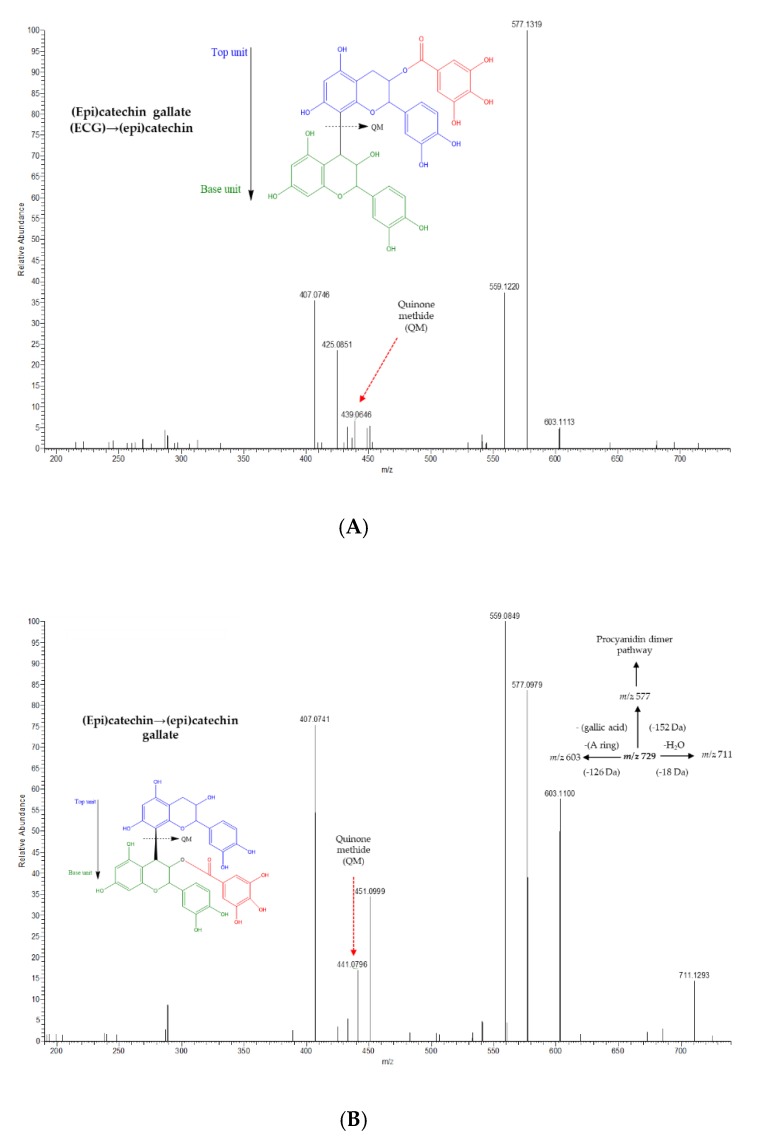
The MS^2^ product ion scan of *m/z* 729 shows key fragment ions at *m/z* 439 and 441 produced by QM fragmentation for (epi)catechin gallate (ECG)→(epi)catechin (**A**) and (epi)catechin→(epi)catechin gallate (**B**), respectively.

Four PAs with gallic acid esters were also tentatively identified by MS^2^ only in the Muscat variety: two (epi)catechin gallate→(epi)catechin (*m/z* 729.1436, *m/z* 729.1432), and two (epi)catechin→(epi)catechin gallate (*m/z* 729.1429, *m/z* 729.1436). Both forms produced ions at *m/z* 577, 559, 425, and 407, which are typical of B-type procyanidin dimers [21,40]. QM fission is a fundamental pathway underlying the gallic acid ester position [21]. Thus, the ions at *m/z* 439 and 441 were crucial in assigning the gallic acid ester to the upper and bottom positions, respectively [21] (Figure 2). (Epi)catechin-(epi)catechin gallate (*m/z* 729) has been previously detected in raisins [32], although the specific monomeric sequence is proposed here for the first time.

Epicatechin gallate (*m/z* 441.0811) was also identified only in Muscat raisins and confirmed by comparison with its standard. The mass spectra of epicatechin gallate showed two fragments from the cleavage of the ester bond at *m/z* 289 and 169 for epicatechin and gallic acid deprotonated moieties, respectively [21]. Epicatechin gallate has been previously identified in raisins [13].

#### 2.1.3. Flavonols, Flavones, and Derivatives

Flavonols are extensively distributed in plants and are present in numerous fruits and vegetables. Human intervention trials with isolated flavonols demonstrated an antihypertensive effect [41].

A total of nine flavonols and one flavone were tentatively identified in the three raisin varieties. Quercetin-3-*O*-glucuronide (*m/z* 477.0658), quercetin-*O*-hexoside isomer 2 (*m/z* 463.0865), kaempferol-*O*-hexoside (*m/z* 447.0917), isorhamnetin-*O*-hexoside (*m/z* 477.1021), and quercetin (*m/z* 301.0341) were detected in all raisin varieties. Quercetin-3-*O*-glucuronide and quercetin-*O*-hexoside showed the same fragment at *m/z* 301, owing to respective losses of glucuronide and hexoside moieties. Moreover, quercetin-3-*O*-glucuronide was confirmed by comparison with its pure standard. Kaempferol-*O*-hexoside produced the deprotonated aglycone fragment at *m/z* 285, suggesting it arose from either luteolin or kaempferol. Additional structural information provided by MS^2^ detection of lower intensity ions allowed the aglycone to be identified as kaempferol, based on the characteristic product ion at *m/z* 255 [42]. Isorhamnetin-*O*-hexoside was tentatively identified by comparison with the mass spectra reported by Simirgiotis et al. [43]. Quercetin was confirmed by comparison with an authentic standard.

Quercetin-*O*-hexoside isomer 1 (*m/z* 463.0864) and kaempferol-3-*O*-glucoside (*m/z* 447.0915) were only detected in MS^2^ mode in Muscat and sultana raisins. Both compounds showed the cleavage of the sugar moiety (−162 Da) generating the ions at *m/z* 301 (quercetin) and *m/z* 285 (kaempferol), respectively. Additionally, kaempferol-3-*O*-glucoside revealed a fragment at *m/z* 255 typical of kaempferol aglyconem, which was confirmed by comparison with a standard.

Rutin (*m/z* 609.1428), kaempferol-*O*-rutinoside (*m/z* 593.1491), and luteolin-*O*-glucuronide (flavone) (*m/z* 461.0710) were only detected in sultanas. The mass spectra of rutin showed ions at *m/z* 301 and 300 due to the loss of the rutinoside unit [M − H—308]^−^, and to the radical anion of the aglycone (*m/z* 300) [44], respectively. Furthermore, rutin was confirmed by comparison with its pure standard. Kaempferol-*O*-rutinoside and luteolin-*O*-glucuronide revealed the same fragment at *m/z* 285 owing to the loss of rutinoside (−308 Da) and glucuronide (−176 Da) moieties, respectively. The characteristic product ions at *m/z* 255 and 175 allowed the identification of the aglycone as well as kaempferol and luteolin, respectively [42]. Luteolin-*O*-glucuronide was the only flavone detected in all varieties. Kaempferol-3-rutinoside has been previously reported in raisins [13].

The number of flavonol derivatives was higher in sultanas, which are produced by immersing grapes in an alkaline potassium carbonate solution before drying [45]. This chemical pretreatment could inactivate polyphenol oxidase, and thus protect the phenolic compounds from degradation.

Several studies have identified and quantified flavonols (e.g., rutin, quercetin-hexoside, quercetin-glucuronide, kaempferol-hexoside, isorhamnetin-hexoside, and quercetin) in raisins [2,5,13], but to the best of our knowledge, this is the first report of luteolin-*O*-glucuronide in these particular varieties.

#### 2.1.4. Flavanones and Derivatives

Flavanones have been widely studied in citrus fruits. They are reported to enhance the defenses of the organism against oxidative stress and help in the prevention of cardiovascular diseases, atherosclerosis, and cancer [46]. Two conjugated flavanones were detected in raisins and identified through their MS spectra.

Hesperidin (*m/z* 609.1802) was detected in all three varieties and confirmed by comparison with its pure standard. Eriodictyol-*O*-hexoside (*m/z* 449.1070) was tentatively identified only in Muscat raisins. The MS^2^ of *m/z* 449 showed a characteristic product ion at *m/z* 287 (eriodictyol) generated by the loss of a hexose moiety (−162 Da). Additionally, eriodictyol-*O*-hexoside was tentatively identified by comparison with the mass spectra of previous studies using the LTQ-Orbitrap to analyze red wine [31] and grape pomace [47]. To the best of our knowledge, this is the first report of eriodictyol-*O*-hexoside in raisins of these varieties, although it has been previously identified in grape skins [48].

#### 2.1.5. Stilbenes and Derivatives

The most well-known stilbene is resveratrol, associated with activity against pathologies related to oxidative stress, inflammatory biomarkers, type 2 diabetes, and cardiovascular and neurological diseases [49]. Oligomer derivatives of resveratrol, which are formed from two to eight or even more resveratrol units, have multiple beneficial properties, and some are superior in activity, stability, and selectivity compared with resveratrol. In particular, hopeaphenol and viniferin present a wide range of preventive or anticancer properties [50].

A stilbenoid tetramer (hopeaphenol or isohopeaphenol) (*m/z* 905.2557) and a stilbenoid dimer (viniferin) (*m/z* 453.1330) were tentatively identified in two raisins varieties. A stilbenoid dimer was present in Muscat and sultana varieties, whereas a stilbenoid tetramer was only detected in sultanas, possibly owing to the production process described above. The MS^2^ of stilbenoid tetramer revealed ions at *m/z* 811 and 717 produced by the loss of one and two phenol moieties, respectively [21]. The MS^2^ of *m/z* 453 showed product ions at *m/z* 359 and 347 produced by the loss of a phenol group (−94 Da) and a 4-methylenecyclohexa-2,5-dienone (−106 Da), respectively [21]. Stilbenoid oligomers have been previously detected in grape canes [21,51], leaves [52], and stems [53], but as far as we know, this is the first time that a stilbenoid tetramer (hopeaphenol or isohopeaphenol) and viniferin have been identified in raisins of these varieties. Stilbenoids are compounds that perform a critical role in the defense mechanism of plants, particularly in the complex response to biotic and abiotic stress [52,54]. Thus, the drying process could provoke stress conditions and stimulate the presence of these phenolic compounds in raisins.

## 3. Materials and Methods

### 3.1. Standards and Reagents

All samples and standards were handled without exposure to light. Gallic, 2-hydroxybenzoic, 4-hydroxybenzoic and caffeic acids (3,4-dihydroxycinnamic acid), (+)-catechin, (−)-epicatechin, quercetin-3-rutinoside (rutin), quercetin-3-*O*-glucuronide, hesperitin-7-rutinoside (hesperidin), and quercetin were purchased from Sigma-Aldrich (St. Louis, MO, USA). Ethylgallate (gallic acid ethyl ester), (−)-epicatechin gallate, and kaempferol-3-*O*-glucoside were acquired from Extrasynthèse (Genay, France).

Folin–Ciocalteau (F–C) reagent was obtained from Sigma-Aldrich (St. Louis, MO, USA). Acetonitrile, water, formic acid, and ethanol were acquired from Merck (Darmstadt, Germany). All solvents were of HPLC grade and all chemicals were of analytical reagent grade. Ultrapure water was obtained from a Milli-Q water purification system (Millipore Bedford, MA, USA).

### 3.2. Extraction of Polyphenols

Commercial raisins (*V. vinifera* L.) of three different types and origins—(i) Thompson seedless (from Chile), (ii) Muscat (from Spain), and (iii) sultanas (from Turkey)—were purchased in a local supermarket in Barcelona, Spain. The extraction was performed following a previously reported procedure with minor modifications [55].

A sample of each variety of raisin (0.5 g, *n* = 3) was homogenized with an ULTRA-TURRAX^®^ (IKA, Staufen, Germany) for 30 seconds, and then vortexed for 1 min with 4 mL ethanol/water (80:20, v/v). Later, they were sonicated in an ultrasound bath (Bandelin electronic GmbH&Co.KG, Berlin, Germany) during 10 min and centrifuged at 4000 RPM for 5 min at 4 °C. The supernatants were collected and the extraction procedure was repeated twice. The supernatants obtained were combined and evaporated with a sample concentrator (MIVAC^®^, FisherScientific, UK) at room temperature under a stream of nitrogen gas, and the residue was reconstituted into 0.1% of aqueous formic acid (4 mL). The raisin extracts were filtered through a 13 mm, 0.45 µm PTFE filter into an amber vial and stored at −20 °C until analysis by LC-LTQ-Orbitrap.

### 3.3. LC-LTQ-Orbitrap-MS Analyses

Liquid chromatography analysis was performed using an Accela chromatograph (Thermo Scientific, Hemel Hempstead, UK) equipped with a quaternary pump, a photodiode array detector (PDA), and a thermostated autosampler. Chromatographic separation was carried out with an Atlantis T3 column 2.1 × 100 mm, 3 µm (Waters, Milford, MA, USA). Gradient elution of analytes was performed with H_2_O/0.1% H-COOH (solvent A) and CH_3_CN (solvent B). The following gradient was used: 0 min, 2% B; 0–2 min, 8% B; 2–12 min, 20% B; 12–13 min, 30% B; 13–14 min, 100% B; 14–17 min, 100% B; and 17–18 min, 2% B, and the column was equilibrated for 5 min to initial conditions [21]. The flow and injection volume were 0.350 mL/min and 5 µL, respectively.

For accurate mass measurements, the LC system was coupled to an LTQ-Orbitrap Velos mass spectrometer (Thermo Scientific, Hemel Hempstead, UK) equipped with an ESI source operating in negative mode. Instrument control and data acquisition were performed with Xcalibur 3.0 software (Thermo Fisher Scientific). The mass range in Fourier transformation mass spectrometry (FTMS) mode was from *m/z* 100 to 1000 [21]. The most intense ions detected in the FTMS spectrum were selected for the data-dependent scan. Parent ions were fragmented by high-energy C-trap dissociation with normalized collision energy of 35 V and an activation time of 10 ms. Operation parameters were as follows: source voltage, 4 kV; auxiliary gas, 10 a.u. (arbitrary units); sweep gas, 2 a.u.; sheath gas, 20 a.u.; and capillary temperature, 275 °C. Raisin samples were analyzed in FTMS mode at a resolving power of 30,000 (full width at half maximum at *m/z* 400) and data-dependent MS^2^ events acquired at a resolving power of 15,000.

### 3.4. Analysis of Total Polyphenols

For the TP assay, each sample was analyzed in triplicate; 24 μL of the raisin extracts was mixed with 184 μL of Milli-Q water in a thermo microtiter 96-well plate (Bio-Rad Laboratories GmbH, Feldkirchen, Germany). Then, 12 μL of F–C reagent (2 N) and 30 μL of sodium carbonate (200 g/L) were added and mixed by vortex following the procedures described previously by our group [55]. The mixtures were incubated for 60 min at room temperature in the dark. After the reaction period, 50 µL of Milli-Q water was added and the absorbance was measured at 750 nm in a microplate reader (Bio-Rad iMark™, Feldkirchen, Germany). The results were expressed as mg of GAE/100 g.

### 3.5. Statistical Analysis

The significance of the results and statistical differences were analyzed using Statgraphics^®^ Centurion (StatPoint, Inc., Warrenton, VA, USA). Analysis of variance was used to compare the means of groups of measurement data, followed by Tukey HSD post-hoc tests, with a significance level of *p* = 0.05.

## 4. Conclusions

The use of LC coupled to LTQ-Orbitrap MS proved to be an efficient and powerful analytical tool to identify a wide range of phenolic compounds in Thompson seedless, Muscat, and sultana raisins. A total of 45 phenolic compounds, namely, hydroxybenzoic (9) and hydroxycinnamic acids (8), flavonols (9), flavones (1), flavanones (2), stilbenes (2), and flavanols (14), were identified or tentatively characterized based mainly on their accurate mass measurements provided by the LTQ-Orbitrap, the fragmentation data from MS^2^ analyses, as well as the mass information obtained from pure standards and the literature. Among the 45 identified phenolic compounds, coumaric acid-*O*-hexoside, ferulic acid-*O*-hexoside, luteolin-*O*-glucuronide, eriodictyol-*O*-hexoside, stilbenoid tetramer (hopeaphenol or isohopeaphenol), and viniferin, to our knowledge, have been identified for the first time in these raisin varieties.

The three types of raisins showed some differences in their phenolic profiles. The most notable difference was the presence of condensed tannins in Muscat raisins, attributed to their seed content. Furthermore, sultanas showed a greater diversity of flavonols. The most commonly detected phenolic compound class in each raisin variety was phenolic acids.

This investigation achieved an exhaustive characterization of the phenolic profile of three widely consumed raisins, which might be helpful for further research on the health properties of distinct raisin varieties.

## Figures and Tables

**Table 1:** Phenolic compounds in raisin extracts tentatively identified by liquid chromatography coupled with electrospray ionization hybrid linear trap quadrupole-Orbitrap mass spectrometry (LC/ESI-LTQ-Orbitrap-MS) in negative mode.

Compounds	Raisins	Accurate Mass [M − H]^−^	MS/MS Ions (% Relative Abundance)	MF
PHENOLIC ACIDS AND DERIVATIVES				
**HYDROXYBENZOIC ACIDS**				
Galloyl-hexoside (1)	M,S	331.0657	169.0133(100), 125.0236(5)	C_13_H_16_O_10_
Gallic acid*	T,M,S	169.0135	125.0237(100)	C_7_H_6_O_5_
Protocatechuic acid-*O*-hexoside (1)	T,M,S	315.0709	153.0184(100), 109.0289(10)	C_13_H_16_O_9_
Galloyl-hexoside (2)	M,S	331.0656	169.0132 (100), 125.0236(5)	C_13_H_16_O_10_
Protocatechuic acid	T,M,S	153.0186	109.0289(100)	C_7_H_6_O_4_
Hydroxybenzoic acid hexoside	M,S	299.0761	137.0238(100), 179.0342(75), 239.0551(70), 209.0449(20)	C_13_H_16_O_8_
2-Hydroxybenzoic acid*	T,M,S	137.0238	93.0340(100)	C_7_H_6_O_3_
Gallic acid ethyl ester (ethylgallate)*	M	197.0446	169.0136(100)	C_9_H_10_O_5_
4-Hydroxybenzoic acid*	T,S	137.0238	93.0340(100)	C_7_H_6_O_3_
**HYDROXYCINNAMIC ACIDS**				
Caftaric acid (1)	M,S	311.0394	149.0079(100), 179.0337(40)	C_13_H_12_O_9_
Dihydroxy cinnamic acid (1)	T,M,S	179.0342	135.0444(100)	C_9_H_8_O_4_
Caftaric acid (2)	T,M,S	311.0395	149.0084(100), 179.0343(20)	C_13_H_12_O_9_
Coutaric acid	T,M,S	295.0447	163.0391(100)	C_13_H_12_O_8_
**Coumaric acid-*O*-hexoside**	T,M	325.0914	163.0390(100)	C_15_H_18_O_8_
Dihydroxy cinnamic acid (2)*	T,M,S	179.0343	135.0444(100)	C_9_H_8_O_4_
Fertaric acid	M,S	325.0550	193.0494(100), 149.0082(10)	C_14_H_14_O_9_
**Ferulic acid-*O*-hexoside**	T,M,S	355.1020	193.0498(100)	C_16_H_20_O_9_
**FLAVONOIDS**				
**FLAVANOLS (PROANTHOCYANIDINS)**				
B-type procyanidin dimer (1)	M	577.1330	425.0853(100), 451.1008(60), 407.0750(50), 289.0699(40), 559.1217(20)	C_30_H_26_O_12_
B-type procyanidin dimer (2)	M	577.1329	425.0852(100), 451.1007(60), 407.0749(50), 289.0698(30), 559.1218(10)	C_30_H_26_O_12_
Catechin*	M,S	289.0707	245.0807(100), 205.0496(40), 179.0342(15),	C_15_H_14_O_6_
B-type procyanidin trimer (1)	M	865.1942	695.1360(100), 739.1622(60), 577.1317(55), 575.1165(30), 713.1459(30), 847.1819(20), 449.0850(20), 407.0750(20), 451.1006(15), 425.0853(15), 287.0540(15)	C_45_H_38_O_18_
B-type procyanidin dimer (3)	M	577.1328	425.0850(100), 451.1006(60), 407.0747(50), 289.0697(30), 559.1215(15)	C_30_H_26_O_12_
B-type procyanidin dimer (4)	M	577.1326	425.0853(100), 451.1009(60), 407.0750(40), 289.0699(30), 559.1221(10)	C_30_H_26_O_12_
Epicatechin*	M,S	289.0705	245.0809(100), 205.0497(40), 179.0343(20)	C_15_H_14_O_6_
(Epi)catechin gallate→(Epi)catechin (1)	M	729.1436	577.1319(100), 559.1220(40), 425.0851(20), 407.0746(35), 439.0646(5), 603.1113(5)	C_37_H_30_O_16_
(Epi)catechin gallate→(Epi)catechin (2)	M	729.1432	577.1312(100), 559.1214(40), 407.0747(30), 425.0850(20), 439.0644(5), 603.1092(5)	C_37_H_30_O_16_
B-type procyanidin trimer (2)	M	865.1944	695.1354(100), 739.1614(60). 577.1322(60), 575.1161(40), 713.1462(40), 407.0743(25), 449.0839(20), 425.0851(20), 847.1826(20), 287.0540(20), 543.0894(15), 451.1001(15), 289.0692(10)	C_45_H_38_O_18_
(Epi)catechin→(Epi)catechin gallate (1)	M	729.1429	559.0857(100), 407.0746(90), 577.0980(85), 603.1100(65), 441.0800(20), 451.1010(35), 711.1313(15)	C_37_H_30_O_16_
(Epi)catechin→(Epi)catechin gallate (2)	M	729.1436	407.0747(100), 603.1107(85), 577.1265(75), 559.0879(65), 451.1012(65), 441.0799(55), 711.1306(25), 425.0859(10)	C_37_H_30_O_16_
B-type procyanidin dimer (5)	M	577.1334	425.0849(100), 451.1003(70), 407.0744(60), 289.0699(30), 559.1204(20), 287.0540(10)	C_30_H_26_O_12_
Epicatechin gallate*	M	441.0811	289.0699(100), 331.0439(25), 169.0132(20), 271.0595(10).	C_22_H_18_O_10_
**FLAVONOLS**				
Rutin (quercetin-3-rutinoside)*	S	609.1428	301.0334(100), 300.0256(30)	C_27_H_30_O_16_
Quercetin-*O*-hexoside (1)	M,S	463.0864	301.0330(100)	C_21_H_20_O_12_
Quercetin-3-*O*-glucuronide*	T,M,S	477.0658	301.0334(100)	C_21_H_18_O_13_
Quercetin-*O*-hexoside (2)	T,M,S	463.0865	301.0337(100)	C_21_H_20_O_12_
Kaempferol-3-*O*-glucoside*	M,S	447.0915	284.0311(100), 285.0387(60), 327.0491(15), 255.0284(5)	C_21_H_20_O_11_
Kaempferol-*O*-rutinoside	S	593.1491	285.0385(100), 255.0284(5)	C_27_H_30_O_15_
Kaempferol-*O*-hexoside	T,M,S	447.0917	284.0311(100), 285.0388(85), 255.0282(10)	C_21_H_20_O_11_
Isorhamnetin-*O*-hexoside	T,M,S	477.1021	314.0410(100), 315.0489(35)	C_22_H_22_O_12_
Quercetin*	T,M,S	301.0341	178.9977(100), 151.0029(60)	C_15_H_10_O_7_
**FLAVONES**				
**Luteolin-*O*-glucuronide**	S	461.0710	285.0387(100), 175.0231(5)	C_21_H_18_O_12_
**FLAVANONES**				
**Eriodictyol-*O*-hexoside**	M	449.1070	287.0546(100), 269.0441(30), 259.0598(30)	C_21_H_22_O_11_
Hesperidin (hesperitin-7-rutinoside)*	T,M,S	609.1802	301.0699(100)	C_28_H_34_0_15_
**STILBENES**				
**Stilbenoid tetramer (Hopeaphenol/Isohopeaphenol)**	S	905.2557	811.2121(100), 717.1716(35)	C_56_H_42_O_12_
**Stilbenoids dimer (viniferin)**	M,S	453.1330	359.0905(100), 347.0903(65)	C_28_H_22_O_6_

T: Thompson; M: Muscat; S: sultana; MF: molecular formula. Isomers are shown in brackets. Fragment ions used for ID are listed in order of relative abundances. *Compounds identified by comparison with pure standards. Compounds (tentatively) identified for the first time in raisins are displayed in bold. All other compounds are tentatively identified using MS/MS data and comparing the fragments found with the literature (see Table S1).

## Supplementary Materials

The following are available online at https://www.mdpi.com/1420-3049/25/6/1281/s1, Table S1: Phenolic compounds tentatively identified using LC-ESI-LTQ-Orbitrap-MS in negative mode: Retention time (min), error (ppm), and reference used for identification [21,33,34,43,47,55,56,57,58,59,60,61].

## Abbreviations

Total polyphenols (TPs); Folin–Ciocalteu (F–C); gallic acid equivalents (GAE); liquid chromatography coupled with an electrospray ionization hybrid linear ion trap quadrupole-Orbitrap-mass spectrometry (LC/ESI-LTQ-Orbitrap-MS); high-performance liquid chromatography (HPLC); mass spectrometry (MS); molecular formula (MF); photodiode array detector (PDA); electrospray ionization (ESI); arbitrary units (a.u.); polytetrafluoroethylen (PTFE); Fourier transformation mass spectrometry (FTMS); proanthocyanidins (PAs); quinone methide (QM); heterolytic ring fission (HRF); retro-Diels-Alder (RDA); (Epi)catechin gallate (ECG).
