# Supplementary material for: A Targeted Approach by High Resolution Mass Spectrometry to Reveal New Compounds in Raisins
Source: Molecules. 2020 Mar 12;25(6):1281. doi: 10.3390/molecules25061281 (PMC7143986; doi:10.3390/molecules25061281)
Supplement: Supplementary file 1 [file molecules-25-01281-s001.pdf]

Supplementary Materials: A targeted approach by high resolution mass spectrometry to reveal new compounds in raisins

Danilo Escobar-Avello, Alexandra Olmo-Cunillera, Julián Lozano-Castellón, María Marhuenda-Muñoz and Anna Vallverdú-Queralt

Table S1. Phenolic compounds tentatively identified using LC-ESI-LTQ-Orbitrap-MS in negative mode: Retention time (min), error (ppm) and reference used for identification.

ID	Compounds	Retention time (min)	Error (ppm)	Reference
**1**	Galloyl-hexoside (1)	2.18	−4.591	[47]
**2**	Gallic acid*	2.44	−4.535	Std.
**3**	Protocatechuic acid-*O*-hexoside (1)	4.72	−3.921	[21]
**4**	Galloyl-hexoside (2)	4.93	−4.138	[47]
**5**	Protocatechuic acid	4.94	−4.783	[56]
**6**	Hydroxybenzoic acid hexoside	5.68	−3.780	[57]
**7**	Caftaric acid (1)	6.22	−4.678	[47,21]
**8**	Dihydroxy cinnamic acid (1)	6.84	−4.759	[55]
**9**	Caftaric acid (2)	6.89	−4.292	[47,21]
**10**	2-Hydroxybenzoic acid*	7.20	−4.505	Std.
**11**	4-Hydroxybenzoic acid*	7.70	−4.870	Std.
**12**	B-type procyanidin dimer (1)	8.72	−3.742	[21,34]
**13**	B-type procyanidin dimer (2)	8.98	−3.949	[21,34]
**14**	Coutaric acid	9.04	−4.272	[21]
**15**	Catechin*	9.34	−3.533	Std.
**16**	Coumaric acid-*O*-hexoside (1)	9.69	−4.493	[56]
**17**	B-type procyanidin trimer (1)	10.02	−4.216	[21,34]
**18**	Dihydroxy cinnamic acid (2)*	10.10	−4.591	Std.
**19**	B-type procyanidin dimer (3)	10.36	−4.019	[21,34]
**20**	Fertaric acid	10.39	−4.508	[34,33]
**21**	B-type procyanidin dimer (4)	10.79	−4.001	[21,34]
**22**	Ferulic acid-*O*-hexoside	11.00	−4.127	[56]
**23**	Epicatechin*	11.53	−4.675	Std.
**24**	Eriodictyol-*O*-hexoside	11.64	−4.241	[47]
**25**	(Epi)catechin gallate→(Epi)catechin (1)	11.92	−3.412	[21,59]
**26**	(Epi)catechin gallate→(Epi)catechin (2)	12.13	−4.605	[21,59]
**27**	B-type procyanidin trimer (2)	12.54	−4.782	[21,60]
**28**	(Epi)catechin→(Epi)catechin gallate (1)	12.65	−4.427	[21]
**29**	Gallic acid ethyl ester(ethylgallate)*	12.68	−4.551	Std.
**30**	(Epi)catechin→(Epi)catechin gallate (2)	12.93	−3.508	[21]
**31**	B-type procyanidin dimer (5)	13.13	−2.996	[21,34]
**32**	Epicatechin gallate*	14.86	−3.083	Std.
**33**	Rutin (quercetin-3-rutinoside)*	15.28	−4.399	Std.
**34**	Quercetin-*O*-hexoside (1)	15.43	−2.701	[60]
**35**	Quercetin-3-*O*-glucuronide*	15.76	−3.466	Std.
**36**	Quercetin-*O*-hexoside (2)	15.78	−3.756	[60]
**37**	Kaempferol-3-*O*-glucoside*	16.61	−2.787	Std.
**38**	Kaempferol-*O*-rutinoside	16.70	−3.512	[47]
**39**	Luteolin-*O*-glucuronide	17.14	−4.314	[61]
**40**	Kaempferol-*O*-hexoside	17.19	−3.947	[58]
**41**	Isorhamnetin-*O*-hexoside	17.47	−4.337	[43]
**42**	Hesperidin (hesperitin-7-rutinoside)*	17.59	−3.798	Std.

**43**	Quercetin*	19.96	−4.172	Std.
**44**	Stilbenoid tetramer (Hopeaphenol/Isohopeaphenol)	20.10	−1.215	[21]
**45**	Stilbenoid dimer (viniferin)	20.46	−1.795	[21]

Compounds are listed in elution order. *Compounds identified by comparison with pure standards (Std.). Isomers are shown in brackets.
